# Supplementary material for: Investigating the active chemical constituents and pharmacology of Nanocnide lobata in the treatment of burn and scald injuries
Source: PLoS One. 2023 Jun 13;18(6):e0287147. doi: 10.1371/journal.pone.0287147 (PMC10263327; doi:10.1371/journal.pone.0287147)
Supplement: Attachment — Submitted filename: Reviwers Comments.docx [file pone.0287147.s004.docx]

**Reviewer's comments**

1. There should be more investigation of chemical composition of different extracts that used in this Study and it is unacceptable to say 'may contains' that repeated for all extracts. You should use advanced method of analysis like GC- MS or HPLC or HPLC MS to be sure about the chemical constituents of the used extracts.
2. What is the time of plant collection?
3. In line 80: Using 15% of the plant extracts. What is your reference about that?
4. Resolution of picture needs to be improved.
5. Tables 5-9: Groups should be rewritten with clarified way.
