# Supplementary material for: Investigating the active chemical constituents and pharmacology of Nanocnide lobata in the treatment of burn and scald injuries
Source: PLoS One. 2023 Jun 13;18(6):e0287147. doi: 10.1371/journal.pone.0287147 (PMC10263327; doi:10.1371/journal.pone.0287147)
Supplement: Attachment — Submitted filename: Response to Reviewers.docx [file pone.0287147.s005.docx]

Review Comments to the Author

Please use the space provided to explain your answers to the questions above. You may also include additional comments for the author, including concerns about dual publication, research ethics, or publication ethics. (Please upload your review as an attachment if it exceeds 20,000 characters)

Dear editor and reviewer,

Thanks for your letter and for the reviewer and editor’ comments concerning our manuscript entitled "Investigating active chemical constituents and pharmacology of *Nanocnide lobata* in the treatment of burn and scald" (Manuscript ID PONE-D-22-27583). We really appreciate *Plos One* for giving us an opportunity to revise our manuscript. The manuscript has been carefully and extensively revised according to the editor and the reviewers’ helpful comments. The changes have been highlighted in blue (reviewer 1#), red (reviewer 2#) and green (editor) of the revised manuscript respectively, all the co-authhave read and approved the revised manuscript.

And the responses were as follows:

Reviewer #1:

1. There should be more investigation of chemical composition of different extracts that used in this study and it is unacceptable to say 'may contains' that repeated for all extracts. You should use advanced method of analysis like GC- MS or HPLC or HPLC MS to be sure about the chemical constituents of the used extracts.

**Response**: Following your suggestion, UPLC-MS experiment has been carried out for clarify the chemical constituents of the used extracts. Please check the details of results in figure 1 and Table 5.

2. What is the time of plant collection?

**Response**: The time of plant collection is April of 2021 which has been added in the manuscript.

3. In line 80: Using 15% of the plant extracts. What is your reference about that?

**Response**: I’m so sorry for the mistake. The sentence of the meaning is “The above 3 parts are mixed evenly with the proportion of 85% white vaseline and 15% extractions (petroleum ether extraction, ethyl acetate ester extraction, and n-butanol extraction) respectively. For the purpose to obtain an ointment of suitable consistency for the screening experiment of active fractions for the further study.” which has been revised in line 81 to line 85.

4. Resolution of picture needs to be improved.

**Response**: Thanks for your careful work. The images of whole manuscript has been improved and re-uploaded as separate files.

5. Tables 5-9: Groups should be rewritten with clarified way.

**Response**: Thank you for your help. The groups have been rewritten with clarified and briefly way. Please check in the table captions part of the manuscript.

Reviewer #2: Reviewer Comments:

1. Authors should rectify the grammar mistake throughout the manuscript.

**Response**: Thank you for your kindly suggestion. The professor Wang of Chongqing Medical and Pharmaceutical College was invited to revised the manuscript. Please check the modified part which highlight in red.

2. Abstract:

Authors frequently used abbreviations in the whole abstract section.

**Response**: Due to the limitation of the magazine on the number of words in the abstract, the author used a lot of abbreviations in the abstract section. Please kindly be informed. Following your advice, the abbreviations was improved. Please check it in line 13 to line 17.

Line#7: Confirm the “colorrections” spelling.

**Response**: Thanks for your careful work. “colorrections” was revised to“color rections” . Please check it in line 7.

3. Introduction:

Line # 57 to 61, Please rewrite the whole sentences because the meaning of these is not clear.

**Response**: We really appreciated your careful work. Following your useful advice. The whole sentences has been revised. Please check it in line 61 to line 65.

4. Methodology section:

Line # 88 to 94, the authors should use a small letter throughout a sentence.

**Response**: The small letter was used throughout the sentence.

In lines # 95 to 97, the authors should use an ELISA test kit.

**Response**: The enzyme linked immunosorbent assay (ELISA) test kit was inserted in the manuscript.

In line # 94, kindly check, whether the sentence is in the present tense or past tense.

**Response**: Thanks for your careful work. The sentence is in past tense which had been revised.

In line # 100, authors should use the first full form then next time use abbreviations.

**Response**: Following your advice, the issues had been solved in the whole manuscript. Please check it in line 105.

5. Result section:

In lines # 182 to 187, the authors should mention Table-1 with these lines.

**Response**: Following your suggestion, the Tables with these lines were provided in the manuscript. Please check it in line 224 to line 230.

In lines # 216 to 221, the authors should rectify the typographic and spelling mistakes.

**Response**: Thanks for your careful work. The sentence lines 280 to 286 had been rectified one by one. Please check it in line 280 to line 286.

6. Discussion section:

In lines # 254 to 256, the authors should complete the sentence (especially neotissue formation and tissue remodeling-----).

**Response**: Thanks for your careful work. The sentence was revised which can be easily understood. Please check it in line 313 to line 315.

In lines # 256 to 259, rephrase the sentence.

**Response**: Following your advice, the sentence was rephrased. Please check it in line 315 to line 317.

In line # 274, explain the local burn wound. The authors should remove the word “local”.

**Response**: The word “local” was removed.

In line # 282, rectify the “revelaed” spellings and check spelling throughout the manuscript.

**Response**: The word “revelaed” spellings was rectified to revealed. Please check it in line 342.

In lines # 298 to 299, mention which active part.

**Response**: The active part (petroleum ether extraction) had been mentioned. Please check it in line 361.

Authors may relate their research work with existing literature.

**Response**: Thanks for your kindly help. Please check the related research work (line 369 to line 377) with refs (line 505 to line 518).

7. Conclusion: the conclusion section should be concise.

**Response**: Following your advice, the conclusion section was rewrite which is concise and brief. Please check it in line 386 to line 394.

^*^**Corresponding author**: Jingxin Mao, Ph. D

School of Pharmacy, Chongqing Medical and Pharmaceutical College, 401331, Chongqing, China.

College of Pharmaceutical Sciences, Southwest University, 400715, Chongqing, China.

Email: [mmm518@163.com](mailto:mmm518@163.com) or [maomao1985@email.swu.edu.cn](mailto:maomao1985@email.swu.edu.cn)

Tel: +86-13752922258
