# Supplementary material for: Investigating the active chemical constituents and pharmacology of Nanocnide lobata in the treatment of burn and scald injuries
Source: PLoS One. 2023 Jun 13;18(6):e0287147. doi: 10.1371/journal.pone.0287147 (PMC10263327; doi:10.1371/journal.pone.0287147)
Supplement: Attachment — Submitted filename: Response to reviewers.docx [file pone.0287147.s006.docx]

PONE-D-22-27583R1

Investigating the active chemical constituents and pharmacology of *Nanocnide lobata* in the treatment of burn and scald injuries

PLOS ONE

Dear Dr. Mao,

Thank you for submitting your manuscript to PLOS ONE. After careful consideration, we feel that it has merit but does not fully meet PLOS ONE’s publication criteria as it currently stands. Therefore, we invite you to submit a revised version of the manuscript that addresses the points raised during the review process.
Dear editor,

Thank you for your letter and comments concerning our manuscript entitled "Investigating the active chemical constituents and pharmacology of *Nanocnide lobata* in the treatment of burn and scald injuries" (Manuscript ID PONE-D-22-27583). We truly appreciate the opportunity to revise our manuscript. The manuscript has been carefully and extensively revised according to your helpful comments. The changes have been highlighted in red in the revised manuscript.

Our responses are as follows:

==============================
ACADEMIC EDITOR:

Dear Authors

As per the reviewers’ comments all amendments have been addressed. The results are well established, but the entire manuscript requires improvements and clarity in English and Scientific language.

**Response**: Following your suggestion, the entire manuscript has been revised by American Journal Experts (https://www.aje.cn/)[.](https://www.aje.cn/.) Please find the Editing Certificate uploaded as an attachment.

Abstract: The section is lacking appropriate scientific language for e.g.The authors have stated “Objective: To analyze the initial chemical constituents of Nanocnide lobata and figure out the effect and active fraction in the treatment of burn and scald”.

**Response**: Thank you for your suggestions. The sentence has been revised to “To identify the most effective fraction of *Nanocnide lobata* in the treatment of burn and scald injuries and determine its bioactive constituents”. Please see lines 2-3 in the manuscript.

What do the authors mean by initial chemical constituents (are they analyzing primary metabolites?) and “figure out the effect” which effect?

**Response**: Thank you for your comments. The authors used two methods to determine the chemical constituents of *Nanocnide lobata.* The basic chemical identification methods applied included a variety of color or precipitation reactions using indicator and chromogenic agents. In addition, UPLC‒MS analyses were utilized to determine specific compounds in *Nanocnide lobata.* The phrase “figure out the effect” was inappropriate and has been deleted from the manuscript. Please see lines 2-3 in the manuscript.

I believe that the Objective statement may be clearer and more understandable and what I perceive from the study, more likely may be stated as “To analyze the most effective fraction of *Nanocnide lobata* in the treatment of burn and scald and determine its bioactive constituents”

**Response**: Thank you for your helpful suggestion. Following your advice, the text has been revised to “To identify the most effective fraction of *Nanocnide lobata* in the treatment of burn and scald injuries and determine its bioactive constituents”. Please see lines 2-3 in the manuscript.

In the methods authors have stated paper assays and test tube assays. I believe that these are not scientific names of the assays, so please add clarity. “Petroleum extraction group” doesn’t depict the proper meaning. “Petroleum extract treated group” is one suggestion or authors may think of a better scientific depiction. What treatment was provided to the control group, model group and positive model group.

**Response**: Thank you for your helpful suggestion. The phrases “paper assays” and “test tube assays” were inappropriate and have been deleted from the manuscript. The text has been changed to the following, which is clear and brief: “Chemical identification methods were used to analyze solutions extracted from *Nanocnide lobata* using petroleum ether, ethyl acetate, n-butanol using a variety of color reactions” Please see lines 4-6 in the manuscript.

In addition, the phrase “petroleum extract-treated group” has been used instead of “petroleum extraction group” throughout the manuscript. Similar revisions to the “ethyl acetate extract-treated group” and “n-butanol extract-treated group” have also been made accordingly.

Which fraction of *Nanocnide lobata* was subjected to UPLC-MS analysis?

**Response**: UPLC‒MS analysis was carried out on the total ethanol extract of *Nanocnide lobata*.

Conclusion shall also be restructured. “This study preliminarily (what do the authors mean by preliminary) identified the type of the chemical compounds and the effect of *Nanocnide lobata* in treatment of burn and scald, which provides the basically foundation for further research on its medicinal value”. This statement doesn’t provide a conclusion of the study.

**Response**: The conclusion has been restructured and the indicated sentence revised. Please see lines 410-419 in the manuscript.

The entire abstract section requires English and scientific language recheck. Many parts are vague and some are mentioned above.

**Response**: We appreciate your helpful suggestion. According to your suggestion, the entire manuscript has been revised by American Journal Experts (<https://www.aje.cn/).> Please find the Editing Certificate uploaded as an attachment.

Similarly Introduction section also requires a thorough recheck

**Response**: The introduction has been revised accordingly.

Line 45: "Long-term fever is prone to various complications and life-threatening" incomplete sentence

**Response**: The indicated sentence has been revised to the following: “Additionally, long-term fever can easily lead to various complications and further endanger life” .Please see lines 50-51 in the manuscript.

Line 53-54: Please recheck

**Response**: The indicated sentence has been revised to the following: “*Nanocnide lobata* is a traditional Chinese medicine (TCM) that is usually used to treat lung heat and cough, scrofula, hemoptysis, burns and scalds, carbuncles, bruises, snakebites, and traumatic bleeding.” Please see lines 59-62 in the manuscript.

Line 56-57. If clinical efficacy has been proven then what are the authors trying to address in the study?

**Response**: We apologize for any misunderstanding. The indicated text has been revised to the following: “*Nanocnide lobata* is commonly used to treat burns and scalds in Chinese folk medicine. However, there has been little modern clinical research on the pharmacology and efficacy of *Nanocnide lobata* in the treatment of burn and scald injuries”. Please see lines 62-65 in the manuscript.

Please recheck Line 65-67.

**Response**: The indicated sentence has been revised to the following: “Chemical identification methods were utilized to investigate the chemical composition of each extracted fraction of *Nanocnide lobata* and provide a chemical foundation for further mechanistic research”. Please see lines 71-73 in the manuscript.

Line 77 -85 are still not clear. The authors have mixed all the fractions and prepared an ointment, then how the authors are creating separate groups of animals with labels “Petroleum extraction group”……..

**Response**: We apologize for any misunderstanding. The indicated sentence has been revised to the following: “Then, the petroleum ether extract, ethyl acetate ester extract, and n-butanol extract were each mixed with white Vaseline at a proportion of 85% white Vaseline and 15% extract to obtain ointments of suitable consistency for the further screening of active fractions.” Please see lines 90-93 in the manuscript.

The preparation of ointment may be moved to methods section. Line 134, 137, 140, 143 headings may be restructured. Also provide references for the phytochemical tests performed for every fraction.

**Response**: Thank you for your suggestion. The description of ointment preparation has been moved to the methods section. In addition, the headings on lines 134, 137, 140, and 143 have been restructured, and references for the phytochemical tests performed for every fraction have been provided.

Line 148: “Preparation of samples” heading may be more appropriate.

**Response**: The indicated heading has been revised to “Preparation of samples”, as suggested. Please see lines 145 in the manuscript.

Section 3.3 also requires restructuring.

**Response**: Section 3.3 has been restructured.

Line 231 and 301, reconsider the headings

**Response**: The headings have been reconsidered and revised.

Discussion: Lines 328, 331-337, 342-348, 360-362, 378-385 require special attention as the scientific and English language are not correct.

**Response**: Thank you for your advice. These sentences have been revised. Please see the detail of discussion part in the manuscript.

Have the authors identified any bioactive compounds from the UPLC-MS, effective in the treatment of burns and scalds. They may mention them in the discussion section.

**Response**: Thank you very much for your suggestion. The bioactive compounds identified by UPLC‒MS that are effective in the treatment of burns and scalds have been mentioned in the discussion section. Please see lines 356-371 in the manuscript.

Conclusion section also requires restructuring.

**Response**: The conclusion section has been restructured accordingly. Please see the detail of conclusion part in the manuscript.

Please recheck the table and figure numbers within text are mentioned appropriately.

**Response**: Thank you for your helpful suggestion. The table and figure numbers have been rechecked and revised as needed.
