# Supplementary material for: Investigating the active chemical constituents and pharmacology of Nanocnide lobata in the treatment of burn and scald injuries
Source: PLoS One. 2023 Jun 13;18(6):e0287147. doi: 10.1371/journal.pone.0287147 (PMC10263327; doi:10.1371/journal.pone.0287147)
Supplement: Attachment — Submitted filename: Review Comments 2nd paper.docx [file pone.0287147.s007.docx]

**Review Report:**

The manuscript entitled **“Investigating the active chemical constituents and pharmacology of Nanocnide lobate in the treatment of burn and scald injuries”** is an interesting study in which authors made attempts to contribute in the field.

**Note: I reviewed the file named (PONE-D-22-27583_R2_Pdf) downloaded from “View Submission” and all my comments are according to the line numberings of the pdf file “PONE-D-22-27583_R2_Pdf”**

**Detailed Comments/Recommendations:**

**Title:**

- Title is well written and clearly indicating the theme of the study.

**Abstract:**

- It will be more appropriate and impressive if the names of any two to three out of 39 compounds are mentioned in line 23.
- In line 8: Kindly add gander of mice as well.
- It will be more impressive if authors may use few numerical results i.e. (TNF-α and IL-10 or TGF-β1) in line 27 and 28.

**Key Words:**

- Three out of five key words are already present in the title. It will be more impressive if the authors use the key words other than the words used in Title.

**Introduction:**

- Overall the introduction is very well written.
- Kindly add family name of Nanocnide lobata anywhere in line 54-56.

**Materials and Methods:**

- Kindly mention the identification/herbarium number, which was obtained after identification of plant from a botanist for future reference at the end of line 80.
- Line 84: Mention the technique used for evaporation of ethanol, involving standard operating conditions eg. Temperature, Pressure etc.
- Line 116: Kindly mention the light and dark duration and standard feeding of animals as well.
- Line 176: Kindly explain the study design clearly for example how much doses of extracts and reference drugs were administered to animals and by which route and for how many days. All study design should be quite clear for the reader. It will be more appropriate and facilitate the reader to understand if the authors mention the dosing of animals in in tabular format.
- Line 203: Kindly mention how much blood was obtained and from which place eg. Heart etc.

**Results & Discussion:**

- Results are well expressed.
- Discussion is well written by the authors.
- Line 357: It will be more appropriate if the authors mentions the importance/advantages of UPLC-MS used here in this study.
- Table 6: Kindly mention the details of control, model and positive drug groups. Give descriptions to facilitate the reader. The table should be self-explanatory. Similarly, Tables 7-10 should also be self-explanatory.
- Figures 3-5 are not explained. What does A, B, C, D, E, and F indicate? Histopathological scoring is missing.
- The authors did not discuss the histopathological results.

**Conclusion:**

- The conclusion is well written.

**References:**

- The authors are advised to review all the references for the strict adherence of the uniformity of Journal’s references style.
- Reference no 13. Lines 478-480: Reference is incomplete. Kindly add on full details.
- Reference no 34. Lines 544-546: Reference is incomplete. Kindly add on full details.
- Check the font style and font size of references as well. It should be uniform.

**Concluding Remarks:**

**The paper needs above mentioned suggestions along with the English Language proof readings by some language expert. Then after fulfilling the corrections the paper will be acceptable.**
