# Supplementary material for: Investigating the active chemical constituents and pharmacology of Nanocnide lobata in the treatment of burn and scald injuries
Source: PLoS One. 2023 Jun 13;18(6):e0287147. doi: 10.1371/journal.pone.0287147 (PMC10263327; doi:10.1371/journal.pone.0287147)
Supplement: Attachment — Submitted filename: Response to reviewers.docx [file pone.0287147.s008.docx]

**Review Report:**

The manuscript entitled **“Investigating the active chemical constituents and pharmacology of Nanocnide lobate in the treatment of burn and scald injuries”** is an interesting study in which authors made attempts to contribute in the field.

**Note: I reviewed the file named (PONE-D-22-27583_R2_Pdf) downloaded from “View Submission” and all my comments are according to the line numberings of the pdf file “PONE-D-22-27583_R2_Pdf”**

Dear editor and reviewer,

Thanks for your letter and comments concerning our manuscript entitled "Investigating active chemical constituents and pharmacology of *Nanocnide lobata*  in the treatment of burn and scald injuries" (Manuscript ID PONE-D-22-27583). We really appreciate *Plos One* for giving us an opportunity to revise our manuscript. The manuscript has been carefully and extensively revised according to your helpful comments. The changes have been highlighted in red in the revised manuscript point by point.

And the responses were as follows:

**Detailed Comments/Recommendations:**

**Title:**

- Title is well written and clearly indicating the theme of the study.

**Response**: Thank you for your help.

**Abstract:**

- It will be more appropriate and impressive if the names of any two to three out of 39 compounds are mentioned in line 23.
- **Response**: Following your suggestion, the names of any two to three out of 39 compounds are mentioned in line 23 to line 25. Please check the detail in the manuscript.
- In line 8: Kindly add gander of mice as well.
- **Response**: Do the reviewer means “gender of mice”? The gender of the mice is female which had been provided in the abstract part of the manuscript. Please check in line 8.
- It will be more impressive if authors may use few numerical results i.e. (TNF-α and IL-10 or TGF-β1) in line 27 and 28.
- **Response**: Thanks for your careful work. Few numerical results were listed in line 29 to line 32.

**Key Words:**

- Three out of five key words are already present in the title. It will be more impressive if the authors use the key words other than the words used in Title.
- **Response**: The key words and title is partly same. However, if I can’t use the similar word in the key words part. It was inability to summarize or indicate the key points of the article well. Therefore, I suggest the key words may changed to “*Nanocnide lobata*, chemical composition, UPLC‒MS, preliminary test, burn and scald injuries, pharmacological effects” .Please check in line 41-42.

**Introduction:**

- Overall the introduction is very well written.]
- **Response**: Thank for your constructive comments.
- Kindly add family name of Nanocnide lobata anywhere in line 54-56.
- **Response**: The family name of *Nanocnide lobata* is *Urticaceae* which was added in the manuscript. Please check it in line 59 to 62.

**Materials and Methods:**

- Kindly mention the identification/herbarium number, which was obtained after identification of plant from a botanist for future reference at the end of line 80.
- **Response**: The original herbarium is kept in the 407 Natural medicinal chemistry Laboratory of the Scientific Research Center of Chongqing Three Gorges Medical College (No.20210503) .Please check it in line 82 to 85.
- Line 84: Mention the technique used for evaporation of ethanol, involving standard operating conditions eg. Temperature, Pressure etc.
- **Response**: Thanks for your careful work. The technique which was used for evaporation of ethanol listed in the manuscript. Please check in line 91.
- Line 116: Kindly mention the light and dark duration and standard feeding of animals as well.
- **Response**: The breeding environment was kept at 25±10℃, the relative humidity was 40% to 70%, and the light-dark cycle was 12 h. Adapt to feeding for consecutive 3 days, fasting 12 h before the experiment, and drinking water freely. Please check in line 123 to 125.
- Line 176: Kindly explain the study design clearly for example how much doses of extracts and reference drugs were administered to animals and by which route and for how many days. All study design should be quite clear for the reader. It will be more appropriate and facilitate the reader to understand if the authors mention the dosing of animals in in tabular format.
- **Response**: Following your suggestion, the the study design has been revised which is clearly and briefly. Please check in line 192 to line 196. In addition, the table 1 titled “Animal grouping and treatment” was added in the manuscript which mention the dosing of animals in in tabular format.
- Line 203: Kindly mention how much blood was obtained and from which place eg. Heart etc.
- **Response**: Take approximately 1 mL of blood from the orbit of mice, deposit for 20 minutes, then centrifuge at 3000 rpm for 5 minutes, and take the upper serum. Store the serum in a refrigerator at -80 ° C for later use. Please check in line 213 to 216.

**Results & Discussion:**

- Results are well expressed.
- **Response**: Thanks for your comments.
- Discussion is well written by the authors.
- **Response**: Thanks for your encouragement.
- Line 357: It will be more appropriate if the authors mentions the importance/advantages of UPLC-MS used here in this study.
- **Response**: The importance/advantages of UPLC-MS used here in this study was presented in the manuscript. Please check it in 376 to 381.
- Table 6: Kindly mention the details of control, model and positive drug groups. Give descriptions to facilitate the reader. The table should be self-explanatory. Similarly, Tables 7-10 should also be self-explanatory.
- **Response**: Thanks for your careful work. The tables 8-11 was revised which mention the details of control, model and positive drug groups.The tables are self-explanatory.
- Figures 3-5 are not explained. What does A, B, C, D, E, and F indicate? Histopathological scoring is missing.
- **Response**: The figures 3-5 has been explained in line 302 to 324, please check. In addition, the histopathological scoring was added in the manuscript. Please check in line 221 to 224 as well as figure 6 (new added).
- The authors did not discuss the histopathological results.
- **Response**: The histopathological results has been discussed in the results part. Please check in line 302 to 329.

**Conclusion:**

- The conclusion is well written.
- **Response**: Thanks for your comments.

**References:**

- The authors are advised to review all the references for the strict adherence of the uniformity of Journal’s references style.
- **Response**: The references style was revised to the style which meet the standards of Plos One.
- Reference no 13. Lines 478-480: Reference is incomplete. Kindly add on full details.
- **Response**: The full details of references style was added.
- Reference no 34. Lines 544-546: Reference is incomplete. Kindly add on full details.
- **Response**: The full details of references style was added.
- Check the font style and font size of references as well. It should be uniform.
- **Response**: The references style was revised one by one which may meet the standards of Plos One.

**Concluding Remarks:**

**The paper needs above mentioned suggestions along with the English Language proof readings by some language expert. Then after fulfilling the corrections the paper will be acceptable.**

**Response**: Following your suggestion, the entire manuscript was revised by American Journal Experts (https://www.aje.cn/)[.](https://www.aje.cn/.) Please check the English and scientific language certificate which uploaded as the attachment.
